# Case Report: Hepatotoxicity Associated with the Use of Hydroxychloroquine in a Patient with COVID-19

**DOI:** 10.4269/ajtmh.20-0276

**Published:** 2020-04-17

**Authors:** Melissa Barreto Falcão, Luciano Pamplona de Góes Cavalcanti, Nivaldo Menezes Filgueiras Filho, Carlos Alexandre Antunes de Brito

**Affiliations:** 1 Research and Extension Center in Health Surveillance, State University of Feira de Santana, Feira de Santana, Brazil;; 2 Department of Community Health, School of Medicine, Federal University of Ceará, Fortaleza, Brazil;; 3 School of Medicine, State University of Bahia, Salvador, Brazil;; 4 School of Medicine, Salvador University, Salvador, Brazil;; 5 Department of Internal Medicine, Clinical Hospital of Federal University, Recife, Brazil;; 6 Tropical Medicine post-graduation of Federal University of Pernambuco, Recife, Brazil;; 7 Autoimmune Research Institute, Recife, Brazil

## Abstract

Hydroxychloroquine (HCQ) has been used for the treatment of novel coronavirus disease (COVID-19) cases. However, evidence of efficacy remains limited, and adverse events can be associated with its use. Here, we report a case of a patient with severe COVID-19 who, after being administered HCQ, exhibited a 10-fold increase in serum levels of transaminases, followed by a rapid decrease after HCQ was withdrawn. Considering the significantly increased use of HCQ during the COVID-19 pandemic, this case alerts us to the potential for HCQ to be associated with hepatotoxicity and the need to monitor liver function during HCQ therapy.

In December 2019, a severe acute respiratory syndrome-coronavirus-2 epidemic was reported in Wuhan, Hubei Province, China. In the months since, the novel coronavirus disease (COVID-19) pandemic has spread to more than 200 countries and caused numerous documented infections.^1–3^

The high incidence and lethality of COVID-19 have caused tens of thousands of deaths. Approximately 20% of identified cases progress to pneumonia requiring hospitalization, and approximately 5% require treatment in intensive care units (ICUs).^4,5^

In the current context of global threat, different therapies are being tested in patients with COVID-19, including antiviral drugs, in an effort to reduce the severity of cases. Among potential therapies, hydroxychloroquine (HCQ) has been used in several countries, despite the limited number of studies published and divergent opinions regarding its use.^6–12^ Importantly, HCQ may be associated with adverse cardiac, ophthalmological, hematological, neurological, musculoskeletal, and gastrointestinal effects, among others.^13–15^

Here, we describe the case of a patient with severe COVID-19 pneumonia who developed hepatotoxicity associated with the use of HCQ, marked by a 10-fold increase in transaminase levels; these levels rapidly regressed following drug withdrawal.

In view of the increased use of HCQ during the COVID-19 pandemic, this report seeks to warn clinical practitioners and policymakers about the potential for HCQ to be associated with hepatotoxicity and the importance of properly monitoring liver function in patients receiving HCQ therapy.

## CASE REPORT

On March 20, 2020, a 29-year-old woman gave birth at term via cesarean delivery. Despite vaginal bleeding in the immediate postpartum period, she soon reached a stable condition with hemoglobin of 7.0 g/dL and was discharged after 2 days. The newborn, weighing 3.900 kg, showed no complications.

On returning home, the patient had contact with her parents, who had recently arrived from the city of Brasilia, Brazil, with symptoms of fever and dry cough. After 5 days of contact, she experienced the first symptoms of the disease, with weakness, dry cough, dyspnea, and an episode of hemoptysis on March 25, 2020.

**Figure 1.**
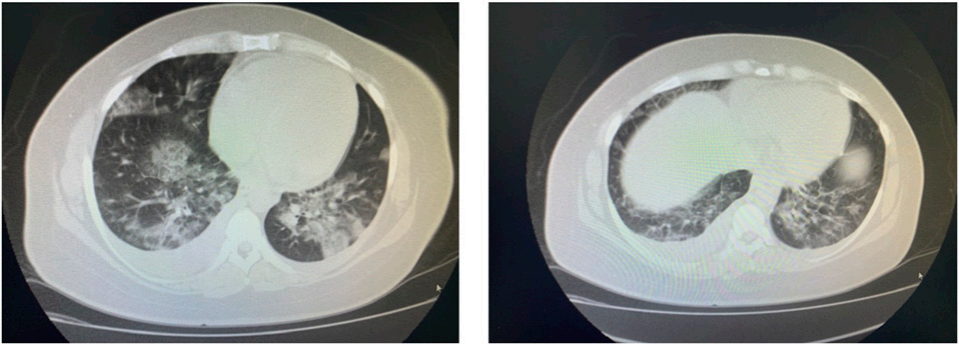
Chest tomography of the patient on day 8 of novel coronavirus disease infection. The presence of bilateral pleural effusion, with filled areas in the air space and confluent, alternating areas of ground-glass attenuation dispersed in the peripheral and central regions of the lungs.

On day 8 post-delivery (March 28), the patient was admitted to the hospital with severe dyspnea. Chest tomography (CT) showed bilateral pleural effusions, air space opacities, and confluent areas of ground-glass attenuation dispersed in the peripheral and central regions of the lungs (Figure 1). Angiotomography did not reveal thromboembolism, and a duplex ultrasound venous and arterial scan of the lower limbs and abdominal tomography showed normal findings. However, the patient exhibited a marked increase in D-dimer (8,446 ng/mL), C-reactive protein (270 mg/L), and lactate dehydrogenase (1,000 IU/L), and the reverse transcription–polymerase chain reaction (RT-PCR) test result for severe acute respiratory syndrome-coronavirus-2 (SARS-CoV-2) via nasal swab was positive.

On day 2 of hospitalization (March 29), the patient’s chest CT showed marked worsening, and after a sharp drop in oxygen saturation requiring mechanical ventilation and supportive measures, she was transferred to the ICU.

**Figure 2.**
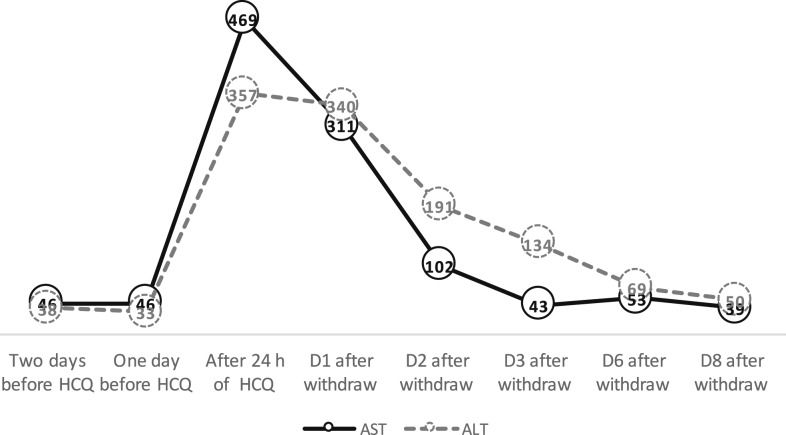
Serum levels of aspartate aminotransferase and alanine aminotransferase in a patient with novel coronavirus disease after the use and withdrawal of hydroxychloroquine. D = day.

At the ICU, on the third day of hospitalization (March 30), the patient received azithromycin and piperacillin–tazobactam. After 3 days in the ICU (day 7 of hospitalization), HCQ at a dose of 400 mg twice per day was prescribed on April 3. One day later, after two doses of HCQ, she exhibited an approximately 10-fold increase in levels of transaminases (Figure 2). Hydroxychloroquine was immediately discontinued, whereas all other medications in use were maintained. Within 5 days after the suspension of HCQ, her levels of transaminases regressed to near-normal values (Figure 2). Serum levels of bilirubin, alkaline phosphatase, and gamma-glutamyl trasferase, and prothombin time and kidney function were normal.

At the time of this article’s submission (April 12), the patient remained in intensive care but showed progressive improvement in clinical, laboratory, and imaging parameters.

The results of RT-PCR tests performed on the patient’s parents, brother-in-law, and newborn were positive. All exhibited mild symptoms.

## DISCUSSION

In the case reported, the patient with acute respiratory distress syndrome due to COVID-19 presented with a rapid increase in transaminases after the introduction of HCQ, followed by a rapid reduction after the drug was discontinued.

Hepatic dysfunction and the elevation of liver enzymes have been reported in 30–60% of cases of COVID-19, more frequently in patients admitted to the ICU, albeit with only slight elevations of liver enzymes.^16–20^ In a study involving 138 hospitalized patients with COVID-19, elevations in transaminases were higher in ICU patients (*P* < 0.001), but with a mean value of 52 U/L and a maximum value of 70 U/L.^16^ In an analysis of 82 deaths caused by COVID-19, levels of enzymes were normal at admission and increased approximately 24 hours before death, often more significantly for AST, with an average of 74.5 U/L and variations from 35.5 to 184 U/L.^21^

In the case reported, the change in liver enzymes did not appear to be due directly to COVID-19. However, we cannot definitively exclude the possibility of other etiologies that may cause hepatic damage in a critically ill patient, such as hypovolemic shock, and the use of other drugs, however, was not observed in this case. The levels of the enzymes were normal in the days before HCQ was introduced and after the drug was withdrawn, and the levels showed a rapid recovery, despite the patient’s persistent severe medical condition, without withdrawal or introduction of other drugs.

Although hepatotoxicity in users of HCQ is uncommon, in some clinical conditions, this risk is higher, including patients using this drug with porphyria cutanea tarda or viral hepatitis.^22–25^ Severe liver dysfunction during the use of HCQ is rare, although it has been documented.^26–29^ Makin et al. reported two cases of patients with rheumatological disease, who, after 2 weeks of using 400 mg of HCQ daily, were admitted with fulminant hepatitis; one required liver transplant, and both patients died.^27^

A rapid normalization of liver enzymes has been described after the withdrawal of HCQ.^28,29^ In another case report, a patient with systemic lupus erythematosus, using 400 mg HCQ daily, had abdominal pain, nausea, vomiting, and diarrhea. There was no evidence of autoimmune disease activity, viral infections were excluded, and the only abnormalities that explained the clinical picture were transaminase elevations alanine aminotransferase 987 U/L and aspartate aminotransferase (AST) 745 U/L. With the withdrawal of the drug, the symptoms regressed and there was a rapid normalization of liver enzymes suggesting drug-induced hepatitis.^28^

In another case, Galvañ et al. reported a patient with mixed connective tissue disease who developed fever, abdominal pain, vomiting, and rapid elevation of liver enzymes, with AST 399 U/L within 10 hours after initiation of HCQ therapy. Symptoms resolved and AST returned to basal levels 5 days after the drug’s withdrawal.^29^

Another factor that may increase the adverse effects of HCQ is the high dose recommended in some protocols for COVID-19. In China, for instance, a described alternative regimen is 500 mg of HCQ twice daily for 7 days, a higher dose than typically used for rheumatological and autoimmune diseases, porphyria cutanea tarda, or malaria prophylaxis, all of which are typically treated with doses of 200–600 mg per day.^10,30^

The mechanisms of hepatic injury related to HCQ are poorly established, and toxicity may be due to reactive metabolites and oxidative stress induced by this drug or an idiosyncratic toxic or synergistic effect associated with inflammatory processes.^26,31,32^ Referring to an experimental rat model, Nikanahad et al. have suggested that concomitant inflammatory processes, including those induced by infections such as malaria, may increase liver damage caused by antimalarial drugs.^32^ Therefore, the potential deleterious synergistic effect of COVID-19 infection and antimalarial drugs needs to be assessed.^33^

Although beyond the scope of this discussion, the case also highlights the high degree of transmissibility of SARS-CoV-2, which reached all family members in a short period, all of whom tested positive according to RT-PCR.

In summary, this case report warns of the potential for hepatotoxicity during the COVID-19 pandemic due to the use of HCQ, which has been administered to patients with COVID-19 in many centers, at varied doses and for those with both mild and severe disease. The case draws attention to the need to reinforce the monitoring of liver function soon after HCQ therapy commences and to maintain monitoring during its use.

## References

[1] Ashour HM Elkhatib WF Rahman MM Elshabrawy HÁ, 2020 Insights into the recent 2019 novel coronavirus (SARS-CoV-2) in light of past human coronavirus outbreaks. Pathogens 9: 1–15. 10.3390/pathogens9030186 PMC7157630 32143502

[2] Gralinski LE Menachery VD, 2020 Return of the coronavirus: 2019-nCoV. Viruses 12: 1–8. 10.3390/v12020135 PMC7077245 31991541

[3] World Health Organization, 2020 Coronavirus Disease 2019 (COVID-19) Situation Report-78. Geneva, Switzerland: World Health Organization.

[4] Wu Z McGoogan JM, 2020 Characteristics of and important lessons from the coronavirus disease 2019 (COVID-19) outbreak in China: summary of a report of 72314 cases from the Chinese center for disease control and prevention. JAMA. [ePub ahead of print 2020 Feb 24]. Available at: 10.1001/jama.2020.2648. 32091533

[5] Mizumoto K Chowell G, 2020 Estimating risk for death from 2019 novel coronavirus disease, China, January–February 2020. Emerg Infect Dis 26: 1–9. 32168464 10.3201/eid2606.200233 PMC7258458

[6] Yao X 2020 In vitro antiviral activity and projection of optimized dosing design of hydroxychloroquine for the treatment of severe acute respiratory syndrome main point: hydroxychloroquine was found to be more potent than chloroquine at inhibiting SARS-CoV-2 in vit. Clin Infect Dis 2: 1–25. 10.1093/cid/ciaa237 PMC7108130 32150618

[7] Liu J 2020 Hydroxychloroquine, a less toxic derivative of chloroquine, is effective in inhibiting SARS-CoV-2 infection in vitro. Cell Discov 6: 1–4. 32194981 10.1038/s41421-020-0156-0 PMC7078228

[8] Gautret P 2020 Hydroxychloroquine and azithromycin as a treatment of COVID-19: results of an open-label non-randomized clinical trial. Int J Antimicrob Agents 2020 Mar 20: 105949 Available at: https://doi:10.1016/j.ijantimicag.2020.105949. 32205204 10.1016/j.ijantimicag.2020.105949 PMC7102549

[9] Cortegiani A Ingoglia G Ippolito M Giarratano A Einav S, 2020 A systematic review on the efficacy and safety of chloroquine for the treatment of COVID-19. J Crit Care 2020 Mar 10. pii: S0883-9441(20)30390-7. Available at: https://doi:10.1016/j.jcrc.2020.03.005. 10.1016/j.jcrc.2020.03.005 PMC7270792 32173110

[10] Liang T, 2020 Handbook of COVID-19 Prevention and Treatment. Available at: https://covid-19.alibabacloud.com.

[11] Keshtkar-Jahromi M Bavari S, 2020 A call for randomized controlled trials to test the efficacy of chloroquine and hydroxychloroquine as therapeutics against novel coronavirus disease (COVID-19). Am J Trop Med Hyg 102: 932–933. 32247318 10.4269/ajtmh.20-0230 PMC7204586

[12] Ferner RE Aronson JK, 2020 Chloroquine and hydroxychloroquine in covid-19. BMJ 2020 Apr 8;369:m1432. Available at: https://doi:10.1136/bmj.m1432. 10.1136/bmj.m1432 32269046

[13] Mégarbane B, 2020 Chloroquine and hydroxychloroquine to treat COVID-19: between hope and caution. Clin Toxicol (Phila) 2020 Apr 2: 1–2. Available at: https://doi:10.1080/15563650.2020.1748194. 10.1080/15563650.2020.1748194 32237918

[14] Mahase E, 2020 Covid-19: six million doses of hydroxychloroquine donated to us despite lack of evidence. BMJ 2020 Mar 23;368:m1166. Available at: https://doi:10.1136/bmj.m1166. 10.1136/bmj.m1166 32205321

[15] Argumánez CM La Fuente ISD Collado ZM Pita DS Gómez BM Izquierdo JAS, 2017 Hydroxychloroquine, a potentially lethal drug. Med Intensiva 41: 257–259. 27480672 10.1016/j.medin.2016.05.004

[16] Wang D 2020 Clinical characteristics of 138 hospitalized patients with 2019 novel coronavirus-infected pneumonia in Wuhan, China. JAMA 323: 1061–1069. 10.1001/jama.2020.1585 PMC7042881 32031570

[17] Guan W 2020 Clinical characteristics of coronavirus disease 2019 in China. N Engl J Med 2020 Feb 28. Available at: https://doi:10.1136/bmj.m1166. 10.1056/NEJMoa2002032 PMC7092819 32109013

[18] Huang C 2020 Clinical features of patients infected with 2019 novel coronavirus in Wuhan, China. Lancet 395: 497–506. 31986264 10.1016/S0140-6736(20)30183-5 PMC7159299

[19] Zhou F 2020 Clinical course and risk factors for mortality of adult inpatients with COVID-19 in Wuhan, China: a retrospective cohort study. Lancet 6736: 1–9. 10.1016/S0140-6736(20)30566-3 PMC7270627 32171076

[20] Chen N 2020 Epidemiological and clinical characteristics of 99 cases of 2019 novel coronavirus pneumonia in Wuhan, China: a descriptive study. Lancet 395: 507–513. 32007143 10.1016/S0140-6736(20)30211-7 PMC7135076

[21] Zhang B 2020 Clinical characteristics of 82 death cases with COVID-19 bicheng. medRxiv 2020.02.26.20028191. Available at: https://doi:10.1017/CBO9781107415324.004.

[22] Al-Bari AA, 2014 Chloroquine analogues in drug discovery: new directions of uses, mechanisms of actions and toxic manifestations from malaria to multifarious diseases. J Antimicrob Chemother 70: 1608–1621. 10.1093/jac/dkv018 PMC7537707 25693996

[23] van Jaarsveld CH 2000 Toxicity of anti-rheumatic drugs in a randomized clinical trial of early rheumatoid arthritis. Rheumatology 39: 1374–1382. 11136881 10.1093/rheumatology/39.12.1374

[24] Sunkara B Roofeh D Silver S Pearson TL Ettel M McCune WJ, 2018 The devil’s in the dosing: severe drug-induced liver injury in a hydroxychloroquine-naive patient with subacute cutaneous lupus erythematosus and porphyria cutanea tarda. Lupus 27: 1383–1386. 29631513 10.1177/0961203318768884

[25] Mok MY Ng WL Yuen MF Wong RWS Lau CS, 2000 Safety of disease modifying anti-rheumatic agents in rheumatoid arthritis patients with chronic viral hepatitis. Clin Exp Rheumatol 18: 363–368. 10895374

[26] Wei CH Penunuri A Karpouzas G Fleishman W Datta A French SW, 2015 Troxis necrosis, a novel mechanism for drug-induced hepatitis secondary to immunomodulatory therapy. Exp Mol Pathol 99: 341–343. 26297838 10.1016/j.yexmp.2015.08.006 PMC4593393

[27] Makin AJ Wendon J Fitt S Portmann BC Williams R, 1994 Fulminant hepatic failure secondary to hydroxychloroquine. Gut 35: 569–570. 8175002 10.1136/gut.35.4.569 PMC1374814

[28] Abdel Galil SM, 2015 Hydroxychloroquine-induced toxic hepatitis in a patient with systemic lupus erythematosus: a case report. Lupus 24: 638–640. 25424894 10.1177/0961203314561667

[29] Galvañ VG Oltra MR Rueda D Esteban MJ Redón J, 2007 Severe acute hepatitis related to hydroxychloroquine in a woman with mixed connective tissue disease. Clin Rheumatol 26: 971–972. 16575495 10.1007/s10067-006-0218-1

[30] Ben-Zvi I Kivity S Langevitz P Shoenfeld Y, 2012 Hydroxychloroquine: from malaria to autoimmunity. Clin Rev Allergy Immunol 42: 145–153. 21221847 10.1007/s12016-010-8243-x PMC7091063

[31] Jamshidzadeh A 2016 Antimalarial drugs-induced hepatic injury in rats and the protective role of carnosine. Pharm Sci 22: 170–180.

[32] Niknahad H Heidari R Firuzi R Abazari F Ramezani M Azarpina N Niknahad H Najibi A Saeedi A, 2016 Concurrent inflammation augments antimalarial drugs-induced liver injury in rats. Adv Pharm Bull 6: 617–625. 28101469 10.15171/apb.2016.076 PMC5241420

[33] Rismanbaf A Zarei S, 2020 Liver and kidney injuries in COVID-19 and their effects on drug therapy; a letter to editor. Arch Acad Emerg Med 8: e17. 32185369 PMC7075271

